# Stem-Cell Niches in Health and Disease: Microenvironmental Determinants of Regeneration and Pathology

**DOI:** 10.3390/cells14130981

**Published:** 2025-06-26

**Authors:** Boris Yushkov, Valerii Chereshnev, Elena Korneva, Victoria Yushkova, Alexey Sarapultsev

**Affiliations:** 1 Institute of Immunology and Physiology, Ural Branch of the Russian Academy of Science, 620049 Ekaterinburg, Russia; b.yushkov@iip.uran.ru (B.Y.);; 2 Institute of Experimental Medicine of the North-West Branch of the Russian Academy of Medical Sciences, 197376 Saint Petersburg, Russia

**Keywords:** stem-cell niche, self-renewal, extracellular matrix, microenvironment, tissue engineering, regenerative medicine, hematopoiesis, angiogenesis, Wnt signaling, extracellular vesicles

## Abstract

Stem-cell behavior is governed not solely by intrinsic genetic programs but by highly specialized microenvironments—or niches—that integrate structural, biochemical, and mechanical cues to regulate quiescence, self-renewal, and differentiation. This review traces the evolution of stem-cell niche biology from foundational embryological discoveries to its current role as a central determinant in tissue regeneration and disease. We describe the cellular and extracellular matrix architectures that define adult stem-cell niches across diverse organs and dissect conserved signaling axes—including Wnt, BMP, and Notch—that orchestrate lineage commitment. Emphasis is placed on how aging, inflammation, fibrosis, and metabolic stress disrupt niche function, converting supportive environments into autonomous drivers of pathology. We then examine emerging therapeutic strategies that shift the regenerative paradigm from a stem-cell-centric to a niche-centric model. These include stromal targeting (e.g., FAP inhibition), which are engineered scaffolds that replicate native niche mechanics, extracellular vesicles that deliver paracrine cues, and composite constructs that preserve endogenous cell–matrix interactions. Particular attention is given to cardiac, hematopoietic, reproductive, and neurogenic niches, where clinical failures often reflect niche misalignment rather than intrinsic stem-cell deficits. We argue that successful regenerative interventions must treat stem cells and their microenvironment as an inseparable therapeutic unit. Future advances will depend on high-resolution niche mapping, mechanobiologically informed scaffold design, and niche-targeted clinical trials. Re-programming pathological niches may unlock regenerative outcomes that surpass classical cell therapies, marking a new era of microenvironmentally integrated medicine.

## 1. Introduction

The idea that a cell’s fate is governed not only by its intrinsic genetic program but also by its microenvironment has deep roots in developmental biology and oncology. A landmark experiment by Spemann and Mangold (1924) showed that the transplantation of the dorsal blastopore lip—the “organizer”—induces an entire secondary body axis in amphibian embryos, proving that local signaling molecules can redirect embryonic cell differentiation [1].

Decades later, microenvironmental control was demonstrated in cancer models. Kinsey’s murine melanoma S-91 proliferated exclusively within lung tissue; when lung parenchyma was implanted subcutaneously, the melanoma grew only in that ectopic lung graft, underscoring the niche’s role in organ-specific metastasis [2,3,4]. Likewise, carcinoma cells injected into blastocysts fail to form tumors, whereas the same cells form teratomas after subcutaneous injection [5,6]. Comparable findings with the Rous sarcoma virus—tumors that arise in the chicken wing but not in the early embryo [5,7,8]—show that the microenvironment can be either tumor-suppressive or tumor-promoting depending on the developmental context [9].

Together, these observations support the core premise of this review: the regenerative capacity, plasticity, and pathological conversion of stem cells are determined as much by their surrounding niche as by the intrinsic properties of the cells themselves. This shift in perspective—from cell-centric to niche-centric—forms the conceptual foundation for modern regenerative medicine.

In mammals, adult stem cells have now been identified in virtually every tissue. They include totipotent, pluripotent, ectodermal, mesenchymal, endodermal, and neural stem cells, as well as stem cells derived from the neural crest, somatic mesenchyme, intermediate mesenchyme, and lateral plate mesenchyme; each category is accompanied by transitional progenitor cells [10,11]. Many of these cell types also circulate in the peripheral blood of adult cats, dogs, sheep, goats, pigs, cattle, and horses [12].

In vitro work confirms that microenvironmental factors decisively bias lineage choice. By supplementing culture media with defined growth factors or small molecules, investigators routinely isolate and expand multipotent mesenchymal stem cells (MSCs) from bone marrow [13] and adipose tissue [14]. These MSCs differentiate not only into mesodermal lineages [15,16] but also into neural [17] and epithelial fates [18], illustrating plasticity that is tightly coupled to external cues.

Within tissues, adult stem cells occupy discrete niches that orchestrate quiescence, self-renewal, and commitment. Some niches—such as those at the base of intestinal crypts or within hair follicle bulges—are architecturally well defined, whereas others, including the post-pubertal mammary gland, prostate, and lung, rely on distributed microenvironmental signals rather than a single anatomic compartment.

Roadmap. Building on these foundational concepts, the present review is organized into a mechanistic four-step hierarchy. Section 2 details the composition and architecture of niches across tissues; Section 3 dissects the molecular signaling axes that animate those structures; Section 4 examines how aging, fibrosis, inflammation, and genetic lesions drive dysregulation and disease conversion; and Section 5 surveys emerging therapeutic strategies—from FAP inhibition to extracellular vesicle delivery—that target the niche itself to enhance regenerative outcomes.

## 2. Composition and Architecture of the Stem-Cell Niche

Stem-cell niches are anatomically discrete microenvironments in which resident stem cells, their stromal neighbors, and a specialized extracellular matrix (ECM) scaffold cooperate to balance quiescence, self-renewal, and lineage commitment. This section establishes the physical blueprint—cells, ECM, and three-dimensional (3D) topology—on which all subsequent signaling and disease processes act.

### 2.1. Cellular Constituents

Immediate stromal neighbors—osteoblasts in bone marrow, fibroblasts in skin, pancreatic telocytes, and other tissue-specific mesenchymal cells—govern stem-cell fate through juxtacrine contacts and paracrine factors [19]. Accessory populations such as endothelial cells, pericytes, macrophages, adipocytes [20,21], mast cells [22], and sympathetic neurons integrate systemic signals with local demands, modulating circadian mobilization or stress-induced recruitment. Vascular sinusoids supply metabolites and cytokines, while autonomic nerve endings synchronize niche activity with whole-body rhythms.

### 2.2. Extracellular Matrix Scaffolds and Mechanics

The ECM provides both a structural lattice and a reservoir of biochemical and mechanical cues. Laminin, collagen, fibronectin, and proteoglycans organize spatial relationships between niche residents, create morphogen gradients, and transmit force. Integrins and cadherins on the stem-cell surface translate ECM stiffness, viscoelasticity, and topography into intracellular signaling cascades that steer proliferation or differentiation [19,23]. In planar epithelia such as the epidermis and intestine, the ECM forms a two-dimensional basement membrane; in neural and bone marrow niches, it assembles into complex 3D trabecular networks that partition oxygen tension and growth factors.

### 2.3. Tissue-Specific Architectural Variants

Although built from similar building blocks, niche architecture diverges dramatically across organs to meet distinct regenerative demands.

- Bone marrow contains an endosteal niche that preserves long-term hematopoietic stem-cell (HSC) quiescence next to trabecular osteoblasts [24,25,26] and a perivascular niche in which two-thirds of HSCs abut CXCL12-rich sinusoids that favor proliferation and short-term progenitor output [27,28,29].
- Dental tissues harbor mesenchymal stem-cell (MSC) pools in pulp, exfoliated deciduous teeth, periodontal ligaments, apical papilla, dental follicle, and oral periosteum; rodents have an additional cervical loop niche that fuels continuous incisor growth [30,31,32,33,34,35,36,37].
- Skin positions epithelial stem cells both at the base of rete ridges in the inter-follicular epidermis and in the hair follicle bulge, the latter of which regenerates the epidermis, follicles, and sebaceous glands after injury [38,39,40].
- Intestinal crypts stack stem cells at the base: symmetric division expands the pool, whereas asymmetric division yields transit-amplifying cells that differentiate while migrating toward villus tips [41,42,43,44].
- Skeletal muscle satellites rest beneath the basal lamina and are poised to repair fibers after mechanical insult [19,45,46].
- The heart lodges putative cardiac stem/progenitor cells (CSCs) in low-stress sub-epicardial zones; cardiomyocytes and fibroblasts form a reciprocal signaling hub that sustains both lineages [47,48,49,50,51,52,53,54,55,56].
- The central nervous system retains neurogenic capacity in the sub-ventricular and sub-granular zones, where neural stem cells (NSCs) interface with vasculature and cerebrospinal fluid [57,58,59,60].
- The testis presents an “open” niche in which spermatogonial stem cells and differentiating progeny share the basal compartment of seminiferous tubules alongside Sertoli cells [61,62,63,64,65].
- The mammary gland alternates between transient terminal-end bud niches in puberty and branching-point reservoirs after lactation; evidence suggests the existence of multiple spatially distinct mammary stem-cell pools along the ductal tree [66,67,68,69].

Collectively, these examples highlight how niche composition, ECM geometry, and tissue mechanics co-evolve to match turnover rate, metabolic load, and biomechanical stress. With the structural and architectural blueprint defined (Table 1), we next dissect the signaling programs that animate these components and coordinate stem-cell behavior.

## 3. Molecular Signaling Axes

### 3.1. A Conserved Pathway Set That Governs Quiescence–Proliferation Balance

Stem-cell self-renewal and lineage specification are regulated by a conserved set of signaling pathways that originate during embryogenesis and remain active throughout adult tissue maintenance. Central among these are the Wnt/β-catenin and Bone Morphogenetic Protein (BMP) pathways, both of which control the balance between quiescence and proliferation across multiple tissue types [70].

In the hematopoietic system, Wnt signaling promotes HSC maintenance and interacts with Notch signaling to support long-term self-renewal [71,72]. Similarly, in the epidermis, Wnt activity is critical for tissue homeostasis and cellular differentiation [73,74,75]. Canonical Wnt/β-catenin signaling also plays a prominent role in rodent dental tissues, where its activation leads to continuous tooth formation, highlighting its importance in epithelial stem-cell regulation [76].

This evolutionarily conserved machinery operates across neurogenic niches as well. In the central nervous system, Wnt signaling—especially when combined with insulin-like growth factor 1 (IGF-1)—enhances neural stem-cell proliferation and post-natal neurogenesis [77]. Conversely, BMPs impose a quiescent state, as seen in the cycling of hair follicle stem cells where Wnt promotes entry into the growth phase while BMP maintains dormancy [40,78,79].

Despite the diversity of tissues, these regulatory systems point to a shared architectural logic: stem-cell differentiation often involves a spatial transition between niches, each with its own unique signaling profile. Stem cells progressively lose contact with their original microenvironment, transitioning into new ones aligned with their lineage commitment [26].

### 3.2. Tissue-Specific Refinements of the Core Pathways

These conserved pathways are further tuned by local cues that reflect each tissue’s functional requirements.

- Intestinal crypt. Paneth cells secrete EGF, TGF-α, Wnt3, and the Notch ligand Delta-like 4 (Dll4), supporting the proliferation and maintenance of Lgr5^+^ intestinal stem cells and preserving epithelial renewal [80,81].
- Neural niche. EGF drives glial lineage commitment by expanding transit-amplifying progenitors, whereas FGF favors neurogenesis in the sub-ventricular zone [82]. IGF-1 from the choroid plexus and endothelium enhances neural stem-cell survival and suppresses apoptosis [83].
- Rodent incisor. Activin, follistatin, BMPs, and FGFs collectively regulate proliferation and differentiation to ensure continuous tooth growth [84].
- Mammary gland. Stem cells integrate Wnt, EGFR, IGFR, RANK, Hedgehog, and Notch inputs to control ductal expansion, alveolar differentiation, and post-lactational remodeling [85].
- Cardiac niche. Soluble ECM-associated factors such as VEGF, TGF-β, and HIF-1α modulate mesenchymal stem-cell behavior after injury; cardiac fibroblasts remodel the ECM and support myocardial repair [66].

### 3.3. Mechanotransduction: ECM as a Signaling Reservoir

Beyond soluble ligands, the extracellular matrix (ECM) supplies biochemical and mechanical signals that interface with canonical pathways. In neural and dental niches, laminin, collagen, and heparan sulfate-rich matrices guide migration and differentiation [85]. Galectins modulate neural progenitor adhesion and motility [86], while dental basal progenitors anchor to the basement membrane via α3β1 and α6β4 integrins that preserve polarity [87].

Through integrin-mediated mechanotransduction, stem cells convert matrix stiffness, viscoelastic relaxation, and topography into intracellular cascades that fine-tune Wnt, Notch, and other pathways, thereby regulating self-renewal, proliferation, and lineage commitment [19,23].

## 4. The Pathological Disruption of the Stem-Cell Niche: From Aging and Inflammation to Organ-Specific Disease

### 4.1. Niche Aging, Stress, and Bidirectional Remodeling

Classic transplantation studies have shown that irradiated bone marrow can still support hematopoiesis, proving that a responsive microenvironment can compensate for stem-cell loss [88,89,90]. However, the regenerative advantage conferred by a healthy niche is gradually lost with age: young hematopoietic stem cells (HSCs) adopt an aged phenotype when exposed to old microenvironments, while aged HSCs restore youthful vigor in hosts [89]. In myeloproliferative neoplasms, this plasticity is further illustrated by the expansion of sinusoidal niches in polycythemia vera and the predominance of endosteal niches in essential thrombocythemia, with therapeutic implications for JAK inhibitor response [91]. Vascular and metabolic stress, exemplified by sickle cell disease, leads to the dramatic remodeling of the bone marrow niche, characterized by tortuous arterioles, fragmented sinusoids, and erythroid–myeloid clusters. Here, reactive oxygen species and HIF-1α induce the up-regulation of VEGF-A, Ang1, Ang2, and VCAM-1, while pathology can be transmitted by the transplantation of diseased marrow and partially reversed by regular transfusions [92].

Importantly, stem cells are not merely passive inhabitants of their niches but actively remodel their microenvironments. Dental pulp stem cells secrete angiogenic factors and form capillary-like structures [93,94,95], while periodontal ligament stem cells are capable of regenerating cementum-like tissue in vivo [32]. In the mammary epithelium, epigenetic modifiers such as HDAC7 can rewire the extracellular matrix (ECM) to maintain stemness, demonstrating a dynamic, bidirectional feedback between niche composition and stem-cell behavior [5,96].

### 4.2. Organ-Specific Failure Modes and Disease Conversion

The dysregulation of the stem-cell niche drives disease across organ systems, with each tissue displaying unique vulnerabilities (Table 2).

For example, in the hematopoietic system, the disorganized vascular niches seen in sickle cell disease impair hematopoiesis and exacerbate anemia. In the reproductive system, defects in the blood–testis barrier or loss of CLDN11 halt spermatogenesis at meiosis, yet the transplantation of spermatogonial stem cells into an intact niche can restore fertility, demonstrating the dominance of the microenvironment over stem-cell genotypes [97,98]. In cardiac tissue, misdirected cues after cell therapy can produce ectopic bone formation (os cordis) or the incomplete coupling of cardiomyocytes, contributing to arrhythmias and contractile dysfunction [99,100,101,102].

**Table 2:** From niche failure to niche-centric therapy.

Tissue	Principal Dysregulation Mechanism	Disease Consequence	Exemplar Restorative Strategy
Bone marrow	ROS/HIF-1α-driven vascular distortion (sickle cell disease)	Ineffective erythropoiesis; anemia	Transfusion-mediated HIF-1α reset; MSC-EV therapy to normalize endothelium [92]
Skin and muscle	Chronic inflammation → FAP^+^ fibroblast expansion	Fibrosis; satellite cell exhaustion	FAP inhibition (vaccines, CAR-T, and small molecules) [103,104,105,106,107,108,109,110,111,112]
Heart	Mis-matched cues after cell therapy	Ectopic calcification; arrhythmia	Composite cell sheet/aggregate grafts that preserve ECM and paracrine balance [113,114,115,116,117,118,119]
Neural	Ageing and neuro-inflammation → ECM degradation	Decline in neurogenesis; cognitive loss	Decellularized 3D scaffolds + IGF-1 delivery (preclinical) [120,121,122,123,124,125,126,127]
Intestine	Dysbiosis/chronic cytokine load	Barrier failure; IBD	EV cocktails containing Wnt + IL-10 to re-program Paneth support (animal models) [128,129]
Testis	Blood–testis barrier collapse (CLDN11 loss)	Meiotic arrest; infertility	Autologous SSC transplantation into an intact niche [97,98]

In the central nervous system, aging and neuroinflammation progressively erode ECM integrity in neurogenic zones such as the SVZ and SGZ, depressing neurogenesis and cognitive plasticity. The intestinal niche is particularly sensitive to chronic inflammation and dysbiosis, which can deplete crypt stem cells and disrupt Paneth cell support, undermining epithelial barrier function [41,42,43,44]. In skeletal muscle, the combination of satellite cell depletion and sustained pro-fibrotic niche signaling accelerates sarcopenia and the development of muscular dystrophy [45]. Mammary gland remodeling after lactation or oncogenic transformation may collapse hierarchical control and increase tumor risk [66,67,68,69]. In dental tissues, inflammatory processes, such as periodontitis and pulpitis, compromise angiogenic and ECM cues and prevent the effective repair of dentin and cementum [93,94,95,96].

Critically, the microenvironment can act as an autonomous driver of disease, independent of stem-cell genotypes. Classic mouse models demonstrate that anemia resulting from intrinsic HSC defects can be cured by healthy marrow, whereas niche-defective mice recover only when the microenvironment itself is restored—affirming that re-establishing a supportive niche is sufficient for hematopoietic recovery [130,131]. In the testis, spermatogonial stem cells from barrier-defective hosts outperform wild-type cells when placed in a healthy niche, while in the heart, transplanted stem cells exposed to mismatched cues may calcify or remain electrically immature, sowing arrhythmogenic substrates [97,98,99,100,101,102]. Collectively, these observations emphasize that organ-specific failures of the niche not only drive pathology but also define the limits and opportunities for regenerative medicine.

## 5. Therapeutic Modulations

### 5.1. The Rationale for Niche-Centered Therapy

Recognizing the niche as both a regulator and a therapeutic target has opened new avenues in regenerative medicine [132]. By modulating microenvironmental cues, investigators can enhance endogenous stem-cell activity, improve the survival of transplanted cells, and convert pathological niches into reparative ones [133]. Recent advances in understanding stem-cell niche dysfunction have revealed that age-related alterations in the microenvironment significantly impair normal homeostatic function and contribute to decreased regenerative capacity [132,133]. Targeting the stem-cell niche microenvironment has proven effective in clinical applications, particularly in mobilizing hematopoietic stem cells through the disruption of normal niche–stem cell crosstalk [132].

Advanced microfluidic systems combined with engineered three-dimensional matrices have provided new insights into stem-cell fate regulation and self-organization, preserving the crucial intercellular interactions and extracellular matrix support that closely mimic natural biological niches [134]. These systems demonstrate enhanced capabilities in promoting angiogenesis and immunomodulation compared to traditional two-dimensional cultures [134]. Gap-junction-mediated communication from the niche has been identified as a critical mechanism controlling stem-cell progeny differentiation, with cAMP transport serving as a key signal [135].

### 5.2. Targeting Stromal Determinants: FAP Inhibition

One prominent target is fibroblast activation protein-α (FAP), a serine protease up-regulated in tumors, fibrotic lesions, and chronic inflammation [136]. The genetic deletion, vaccination, and pharmacologic inhibition of FAP suppress tumor growth and reshape the tumor microenvironment in preclinical models [103,104,105,106,107,108,109,110,111].

Recent developments in FAP inhibitor (FAPI) radiopharmaceuticals have demonstrated remarkable clinical promise, with FAPI-PET imaging showing higher sensitivity than traditional FDG-PET in detecting primary tumors and metastases [137]. Clinical studies have shown that FAP-targeted radioligand therapy is safe and effective for treating patients with progressive metastatic tumors, with 90Y-FAPI-46 therapy controlling disease progression in nearly half of patients with advanced sarcomas [138]. Notably, FAP inhibition has shown therapeutic potential beyond oncology, with studies demonstrating that FAP inhibition promotes cardiac repair by stabilizing brain natriuretic peptides (BNPs) [139,140].

### 5.3. Cytotherapy That Recreates Natural Niche Interactions

Therapeutic strategies increasingly combine stem cells with supportive stromal cells to mimic in vivo interactions [141]. The co-transplantation of MSCs with hepatic stellate cells, for instance, accelerates liver regeneration and improves functional recovery after injury [113,114,115,116,117,118,119,142,143].

Cell sheet engineering and aggregate-based delivery represent scaffold-free approaches that preserve endogenous ECM, enhance transplant survival, and maintain tissue-mimetic architecture [144]. Recent advances in cell sheet engineering have significantly evolved over the past two decades, with improved cell sheet harvesting systems introduced using temperature-responsive surfaces to overcome the limitations of conventional cell harvesting methods [145]. MSC sheets have proven effective in bone repair and implant osseointegration [119,146,147], while micromass aggregates outperform single-cell suspensions in regenerative potency [114,115,116,117,118].

Anchored cell sheet engineering has emerged as a novel scaffold-free platform designed for in vitro modeling, providing precise control over cellular behavior, especially ECM production and remodeling [148,149]. This versatile platform is compatible with other cell sheet engineering techniques and allows different tissues to be linked via fluidic systems for body-level simulations. Scaffold-free three-dimensional culture methods have demonstrated enhanced potential in stem-cell-based therapies, circumventing the use of exogenous biomaterials while preserving crucial intercellular interactions.

### 5.4. Scaffold Engineering and Microenvironmental Design

Re-building a functional niche often requires a biomimetic scaffold [120,121,122,123,150,151]. Decellularized matrices from allo- or xenografts maintain their native ultrastructure and have embedded signals that support stem-cell adhesion, proliferation, and lineage commitment [120]. Taylor et al. showed that whole-heart decellularized scaffolds preserve valves and papillary structures, although pump function remains low (~2% of native power) [121]. Recent developments in decellularized extracellular matrix (dECM) scaffolds have demonstrated their superiority over synthetic alternatives due to their bioactivity and preservation of native architecture [152,153,154].

Advances in biomimetic scaffold development have focused on creating hybrid scaffolds that offer both physical and chemical cues to cells [155]. Digital light processing and 3D printing have enabled the fabrication of radially graded scaffolds that closely replicate the hierarchical structure of native bone tissue [156]. Protein-based bioactive coatings have evolved to combine multiple approaches, creating nanoarchitectonic systems that provide enhanced cellular responses [157,158].

Other strategies harness the fibrotic capsules that form around implants, repurposing them as autologous niches for organ repair [122,123,124,125]. Success has been reported in the skin, vascular, urologic, and skeletal applications. Bioceramics, resorbable polymers, and bioactive coatings further augment angiogenesis, osteogenesis, and controlled factor release [126,159,160,161,162,163]. The clinical applications of bioceramics have registered successful use in triggering soft tissue regeneration in both healthy and diabetic patients, with bioactive glass nanofibers showing particularly promising results [164,165]. Advanced manufacturing techniques, particularly additive manufacturing, have enabled precise control over material macro- and micro-structures, offering new possibilities for resorbable composite scaffolds.

### 5.5. Cell-Free Approaches: Extracellular Vesicle Therapy

Among the emerging modalities, MSC-derived extracellular vesicles (EVs) are gaining momentum as scalable, low-immunogenic alternatives to whole-cell transplantation [166,167]. EV cargo (proteins, RNAs, and lipids) modulates immunity, promotes tissue repair, and influences endogenous stem-cell behavior [168]. Preclinical studies show that EVs enhance fracture healing, exert epigenetic regulation, and provide potent immunomodulation [169,170]. By delivering paracrine signals without live-cell engraftment, EVs offer a practical route to microenvironmental re-programming [171].

Recent comprehensive analyses have revealed that MSC-derived EVs contain over 1900 distinct proteins and numerous regulatory RNAs, with their composition varying significantly based on the tissue source and culture conditions available [172].

The clinical translation of MSC-EVs has progressed rapidly, with multiple ongoing clinical trials evaluating their efficacy in diverse conditions, including COVID-19, ischemic stroke, and osteoarthritis [173]. The immunomodulatory properties of EVs are particularly noteworthy, as they can modulate both innate and adaptive immune responses, promoting the transition from pro-inflammatory to pro-regenerative environments. Engineered EVs loaded with therapeutic miRNAs have shown enhanced efficacy, with specific miRNA clusters demonstrating synergistic effects in promoting tissue repair and reducing cellular apoptosis.

## 6. Conclusions

Adult stem-cell niches are active, adaptive ecosystems in which structural, biochemical, and mechanical cues converge to guide cell fate. Mounting evidence shows that stem cells, far from being passive residents, continually remodel their own microenvironment—adjusting their extracellular matrix composition, cytokine gradients, and vascular architecture to meet physiological demand. When aging, hypoxia, fibrosis, and chronic inflammation distort these cues while the niche itself becomes a driver of pathology: sickle cell bone marrow develops tortuous arterioles and fragmented sinusoids that are reversed by the transfusion-mediated normalization of HIF-1α signaling [92]; similar microenvironmental determinants govern repair in the dental pulp and periodontal tissues [93,94], dictate fertility through an intact or disrupted blood–testis barrier [98,99], and even influence cardiac electrophysiology, where the incomplete niche integration of transplanted cardiomyocytes predisposes to arrhythmias [100,101,102,103]. Because these failures arise even when the intrinsic stem-cell potential remains intact, the niche—not the cell—emerges as the primary therapeutic target.

This field continues to face unresolved challenges across multiple niche types. Cardiac stem-cell identity remains disputed, with c-kit^+^/Sca-1^+^ cells showing variable regenerative capacity and unclear integration into the myocardial syncytium [174,175,176,177,178]. MSC therapies encounter niche-dependent risks such as proarrhythmic potential via connexin-43 disruption and ectopic differentiation in non-skeletal tissues, especially in aged or diabetic environments with impaired CXCL12 signaling [179,180,181,182]. Cancer stem cells (CSCs) hijack stromal signaling to evade immunity and support metastasis, particularly in breast and pancreatic cancers, where CSC–fibroblast crosstalk via IL-6 and FAP plays a critical role [183,184,185].

Regenerative medicine is, therefore, shifting from a cell-centric to a niche-centric paradigm. Promising interventions already span stromal cell targeting with FAP inhibitors [104,105,106,107,108,109,110,111], architectural mimicry through decellularized or bio-inspired scaffolds that deliver appropriate mechanical and biochemical cues, paracrine re-programming via MSC-derived extracellular vesicles, and composite cell sheets or aggregates that preserve endogenous cell–cell and cell–matrix contacts. Recent advances in microfluidic platforms and single-cell analytics now enable the real-time dissection of niche signaling dynamics, while extracellular vesicles offer a lower-risk alternative to direct cell transplantation, reducing tumorigenicity in preclinical models [186]. Pioneering transcription factors such as SOX9, vitamin A–retinoic acid signaling, and the mechanical thresholds revealed by biomimetic scaffolds are emerging as key regulators of adult niche plasticity [187,188,189].

Progress now hinges on three fronts: first, high-resolution single-cell and spatial-omics atlases must chart niche states across lifespans and disease, distinguishing reversible from irreversible failure modes; second, scaffold design and pharmacologic discovery should embed mechanobiology and metabolism, two under-explored but powerful regulators of niche function; and third, rigorously powered clinical trials are needed to establish the safety, durability, and cost-effectiveness of niche-targeted interventions. Additionally, resolving fundamental ambiguities—such as the functional identity of cardiac stem cells, the niche-specific behavior of MSCs, and the origin and support networks of CSCs—will be crucial in tailoring regenerative strategies to disease contexts. By treating stem cells and their microenvironment as an inseparable therapeutic unit, regenerative medicine can achieve predictable, tissue-specific, and lasting repair that surpasses cell-centric approaches alone, heralding a new era of microenvironmentally integrated therapy.

## Figures and Tables

**Table 1:** Blueprint of major adult stem-cell niches.

Tissue (Representative Niche)	Core Cellular Constituents	ECM/Mechanical Hallmark	Dominant Signaling Axes	Primary Homeostatic Role
Bone marrow (endosteal and perivascular)	Osteoblasts, sinusoidal endothelial cells, CAR cells, LepR^+^ MSCs, macrophages	3D trabecular matrix; oxygen and CXCL12 gradients	Wnt ↔ BMP, Notch, Tie2/Ang-1	Balance quiescence vs. rapid hematopoietic output
Intestinal crypt	Lgr5^+^ stem cells, Paneth cells, pericryptal myofibroblasts	2-D basement membrane; steep Wnt/BMP gradient	Wnt3, Dll4/Notch, EGF, BMP	Continuous epithelial renewal
Skin (hair follicle bulge)	K15^+^ bulge stem cells, dermal papilla fibroblasts, melanocyte progenitors	Flexible basement membrane; low stiffness	Wnt/Shh, BMP antagonists	Cyclic hair regeneration and wound repair
Neural (SVZ/SGZ)	GFAP^+^ NSCs, endothelial cells, ependymal cells, microglia	Laminin-rich fractal matrix; CSF contact	FGF, EGF, IGF-1, Wnt, BMP	Adult neurogenesis and cognitive plasticity
Skeletal muscle (satellite)	Pax7^+^ satellite cells, FAPs, macrophages, endothelial cells	Sub-laminar niche; rapid viscoelastic relaxation	HGF/c-Met, FGF2, Notch, Wnt	Myofiber repair and hypertrophy control
Heart (sub-epicardial CSC niche)	c-Kit^+^/Sca1^+^ CSCs, cardiomyocytes, fibroblasts, vSMCs	Low-stress ECM; anisotropic stiffness	VEGF, TGF-β, HIF-1α, Wnt	Paracrine support and limited cardiomyocyte turnover
Testis (basal SSC niche)	SSCs, Sertoli cells, peritubular myoid cells	Open niche; type IV collagen–rich BM	GDNF/RET, FGF2, CSF-1	Continuous spermatogenesis
Mammary gland (branching points/TEB)	Basal MaSCs, luminal progenitors, fibroblasts, adipocytes, macrophages	Dynamic ECM remodeling during cycles	Wnt, RANKL, EGFR, Hedgehog, Notch	Ductal elongation and alveolar expansion

## Data Availability

Not applicable.
